# Reactive myelopoiesis and FX-expressing macrophages triggered by chemotherapy promote cancer lung metastasis

**DOI:** 10.1172/jci.insight.167499

**Published:** 2023-05-08

**Authors:** Caijun Wu, Qian Zhong, Rejeena Shrestha, Jingzhi Wang, Xiaoling Hu, Hong Li, Eric C. Rouchka, Jun Yan, Chuanlin Ding

**Affiliations:** 1 UofL Health - Brown Cancer Center and; 2 Department of Microbiology and Immunology, University of Louisville School of Medicine, Louisville, Kentucky, USA.; 3 Department of Computer Science and Engineering, University of Louisville J.B. Speed School of Engineering, Louisville, Kentucky, USA.; 4 Department of Surgery, Division of Immunotherapy, UofL Health - Brown Cancer Center, University of Louisville School of Medicine, Louisville, Kentucky, USA.

**Keywords:** Immunology, Breast cancer, Cellular immune response, Macrophages

## Abstract

Several preclinical studies have demonstrated that certain cytotoxic drugs enhance metastasis, but the importance of host responses triggered by chemotherapy in regulating cancer metastasis has not been fully explored. Here, we showed that multidose gemcitabine (GEM) treatment promoted breast cancer lung metastasis in a transgenic spontaneous breast cancer model. GEM treatment significantly increased accumulation of CCR2^+^ macrophages and monocytes in the lungs of tumor-bearing as well as tumor-free mice. These changes were largely caused by chemotherapy-induced reactive myelopoiesis biased toward monocyte development. Mechanistically, enhanced production of mitochondrial ROS was observed in GEM-treated BM Lin^−^Sca1^+^c-Kit^+^ cells and monocytes. Treatment with the mitochondria targeted antioxidant abrogated GEM-induced hyperdifferentiation of BM progenitors. In addition, GEM treatment induced upregulation of host cell–derived CCL2, and knockout of CCR2 signaling abrogated the pro-metastatic host response induced by chemotherapy. Furthermore, chemotherapy treatment resulted in the upregulation of coagulation factor X (FX) in lung interstitial macrophages. Targeting activated FX (FXa) using FXa inhibitor or *F10* gene knockdown reduced the pro-metastatic effect of chemotherapy. Together, these studies suggest a potentially novel mechanism for chemotherapy-induced metastasis via the host response–induced accumulation of monocytes/macrophages and interplay between coagulation and inflammation in the lungs.

## Graphical abstract

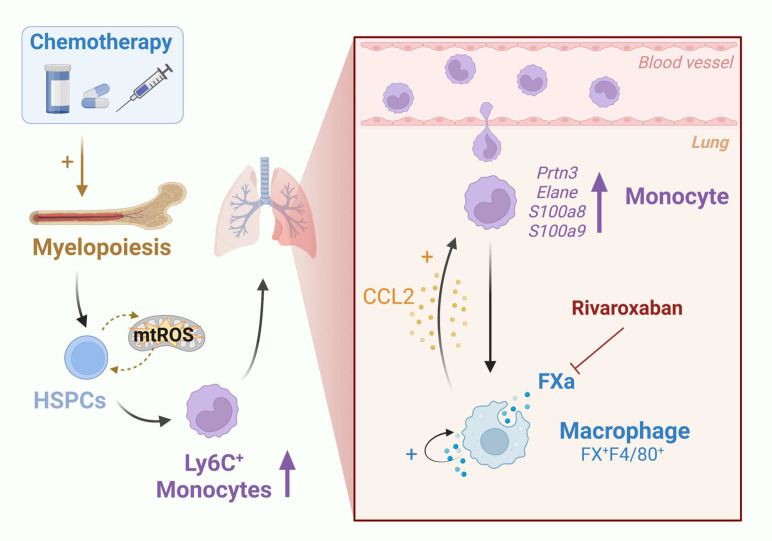

## Introduction

Metastasis is the primary cause of death in patients with breast cancer. The lungs are the second most common site of breast cancer metastasis after the bones (1). Lung metastasis-associated macrophages (MAMs) play very important roles in tumor metastasis through the formation of a pre-metastatic niche (PMN) (2–6). In mouse models of lung metastasis, interstitial macrophages (IMs, CD11b^hi^F4/80^+^CD11c^−^) markedly accumulate in the lungs and differentiate into MAMs (3). Regarding the origin of IMs/MAMs, both tissue-resident macrophages (CCR2^−^) and BM-derived classical monocytes (CCR2^+^Ly6C^+^) contribute to the pool of MAMs (7, 8). Several molecules, such as VEGFR1 (6), MMP1 (9), and TGF-β (10), have been identified to contribute to the pro-metastatic effects of lung macrophages. However, current strategies targeting macrophage-associated molecules have shown limited success in clinical settings (3).

Chemotherapy offers long-term clinical benefits to many patients with cancer. However, several preclinical studies have demonstrated that certain cytotoxic drugs enhance metastasis by multiple mechanisms (11–14). These studies have mainly focused on tumor cell–derived cytokines, chemokines, and exosomes (14–17). Our recent studies suggest that gemcitabine (GEM) promotes accumulation and immunosuppressive function of monocytic myeloid-derived suppressor cells (M-MDSCs) in the tumor microenvironment via the tumor cell–derived GM-CSF and efferocytosis signaling (18). Emerging evidence indicates that the host response induced by chemotherapy may also play a critical role in regulating tumor progression and metastasis (19). This mechanism may explain why tumor recurrence and metastatic rates are still high in cancer patients after primary tumor surgical removal and/or using chemotherapy. However, the critical roles of chemotherapy-induced host responses in promoting metastasis have not been well understood.

In this study, we showed that GEM treatment promoted breast cancer lung metastasis in a spontaneous breast cancer mouse model with accumulated CCR2^+^ monocytes and macrophages in the lungs. Interestingly, increases of CCR2^+^ macrophages and monocytes were also observed in tumor-free mice after GEM and combination of paclitaxel (PTX) and doxorubicin (DOX) treatment. These changes were largely caused by chemotherapy-induced reactive myelopoiesis and upregulation of host cell–derived CCL2. In addition, GEM and combination of PTX and DOX treatment resulted in upregulation of coagulation factor X (FX) in lung macrophages. Inhibition of activated FX (FXa) reduced the pro-metastatic effect of the host response triggered by chemotherapy. These findings support our hypothesis that host responses triggered by chemotherapy enhance breast cancer lung metastasis via modulation of lung macrophage accumulation and differentiation toward a pro-metastatic phenotype.

## Results

### GEM chemotherapy promotes breast cancer lung metastasis and accumulation of lung macrophages.

**Figure 1:**
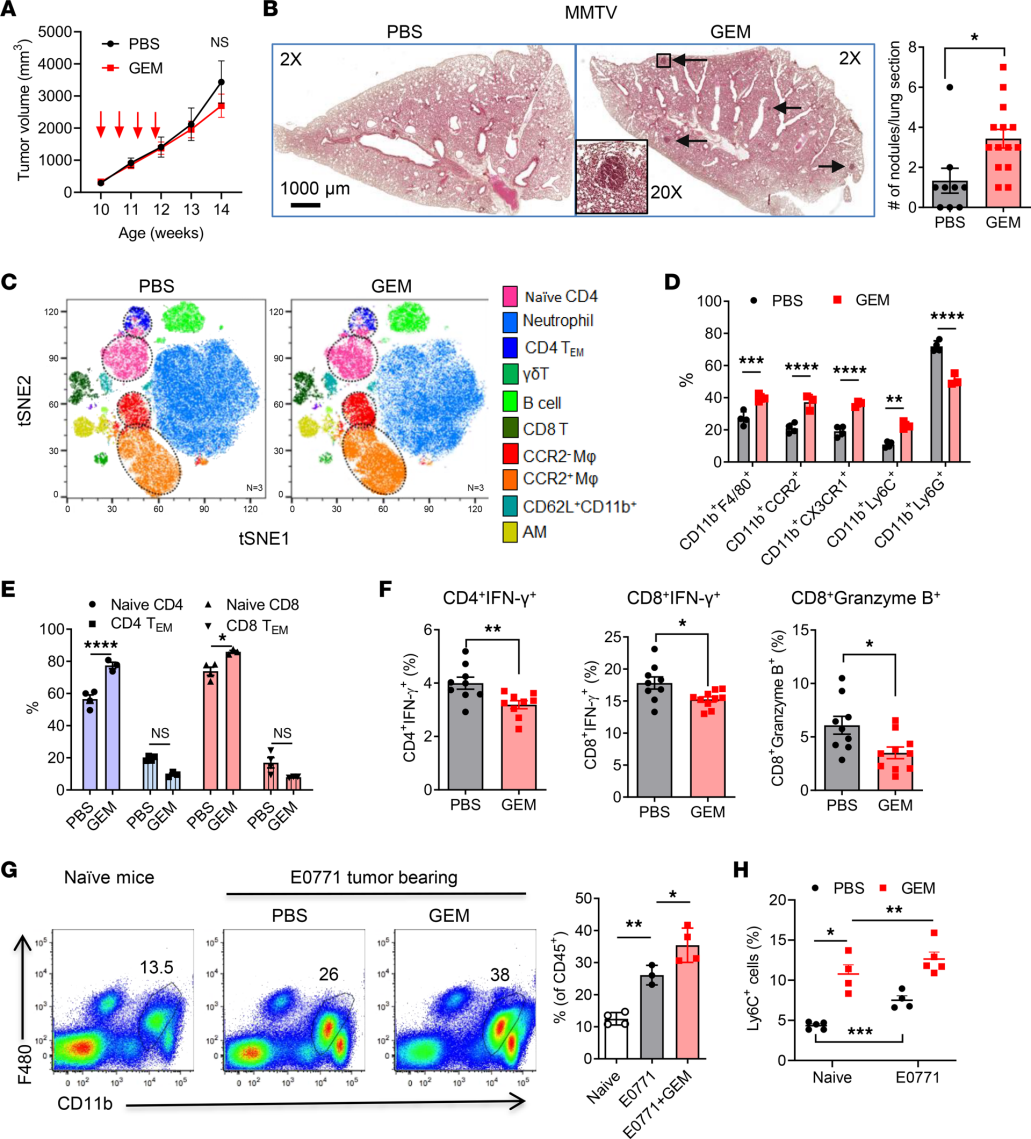
Gemcitabine chemotherapy promotes breast cancer lung metastasis and accumulation of lung macrophages. (**A**) Primary tumor progression after 4 doses of GEM treatment (60 mg/kg, IP). The volume of multiple tumors in each MMTV/PyMT mouse was recorded (*n* = 5–6). (**B**) Representative H&E images of lung sections of MMTV-PyMT mice from PBS- and GEM-treated mice. Number of nodules per lung section was summarized (*n* = 9–14). (**C**) Lung tissues were collected 2 days later. t-Distributed stochastic neighbor embedding (tSNE) plot of major immune cell subsets in the lungs of MMTV-PyMT mice identified by FlowSOM clustering algorithm (gated on CD45^+^). AM, alveolar macrophages. (**D**) Summarized data of major myeloid cells from lungs of PBS- and GEM-treated MMTV-PyMT mice (*n* = 4). (**E**) Summarized data of naive and effector memory CD4^+^ and CD8^+^ T cells in MMTV-PyMT mice (*n* = 4). (**F**) IFN-γ–producing T cells and granzyme B–expressing CD8^+^ T cells in MMTV-PyMT mice were evaluated by intracellular staining and flow cytometry (*n* = 9–10). Each dot represents 1 mouse. (**G** and **H**) E077 tumor–bearing mice (tumor size around 6–8 mm in diameter) were treated by 4 doses of GEM in 2 weeks. Lung macrophages (**G**) and monocytes (**H**) in naive and tumor-bearing mice were analyzed by flow cytometry (*n* = 3–5). Data are representative of 2 or 3 independent experiments and presented as mean ± SEM. *P < 0.05, **P < 0.01, ***P < 0.001, and ****P < 0.0001 by 2-way ANOVA (**A**), ordinary 1-way ANOVA (**D**, **E**, **G**, and **H**), or unpaired 2-sided *t* test (**B** and **F**).

Previous studies have shown that certain chemotherapeutic drugs, such as PTX (14, 20) and DOX (20), promote cancer metastasis in preclinical models. We further examined the effects of GEM on lung metastasis because these drugs exhibit different mechanisms of action. MMTV-PyMT mice develop spontaneous mammary tumors that closely resemble the progression and morphology of human breast cancers with poor prognosis (21). The treatment was started at 9–10 weeks of age, when the biggest single tumor size reached 6–8 mm in diameter, and lasted for 2 weeks. Tumor progression was evaluated for an additional 10–14 days after last treatment. Although GEM treatment did not impact the primary tumor progression (Figure 1A), mice that received GEM treatment developed more lung metastases than those that received PBS control as evidenced by increased tumor nodule counts (Figure 1B). To uncover potential mechanisms underlying the pro-metastatic effect of GEM treatment, the lung immune cell profile was examined 2 days later, after the last GEM treatment, using mass cytometry (CyTOF). The cell clustering analysis revealed a significant increase of lung macrophages, particularly CCR2^+^ macrophages (Figure 1C). CCR2^+^ macrophages are mainly differentiated from BM-derived monocytes (7, 8). Mass cytometry data also showed an increase of CCR2^+^Ly6C^+^ monocytes in the lungs of GEM-treated mice (Figure 1D). Further, we examined the T cell profile in the lungs. GEM treatment significantly increased percentages of naive CD4^+^ and CD8^+^ T cells (CD44^−^CD62L^+^), whereas effector memory T cells (CD44^+^CD62L^−^) were decreased (Figure 1E). Intracellular staining revealed that effector T cells, including IFN-γ–producing CD4^+^/CD8^+^ T cells and granzyme B–expressing CD8^+^ T cells, were significantly decreased in the GEM-treated mice (Figure 1F).

We further addressed the effects of chemotherapy on myeloid cells in the lungs using E0771 tumor–bearing mice. E0771 is characterized between luminal B and triple-negative subtypes and is sensitive to various cancer therapies (22). Our previous studies have shown that multidose GEM treatment results in a reduction of primary tumor progression but increase of Ly6C^+^ myeloid cells in the primary tumor microenvironment (18). We further examined the effects of chemotherapy on lung myeloid cells and found that GEM treatment significantly increased the accumulation of lung MAMs (CD11b^hi^F4/80^+^CD11c^−^) and monocytes (CD11b^hi^Ly6C^+^Ly6G^−^) in E0771 tumor–bearing mice after GEM treatment (Figure 1, G and H). These data suggest that host responses triggered by chemotherapy might contribute to modulation of myeloid cells in lung tissues. We further characterized and compared T cells presented in the lungs of chemotherapy-treated mice. The remarkable decrease of IFN-γ–producing CD4^+^ and CD8^+^ T cells was observed in the lungs of E0771 tumor–bearing mice that received GEM treatment (Supplemental Figure 1; supplemental material available online with this article; https://doi.org/10.1172/jci.insight.167499DS1). Together, these data suggest that a certain chemotherapy treatment may promote lung metastasis through the recruitment of CCR2^+^ macrophages and monocytes into the lungs and subsequent impaired T cell function.

### Chemotherapy-triggered host responses promote tumor metastasis in mice.

**Figure 2:**
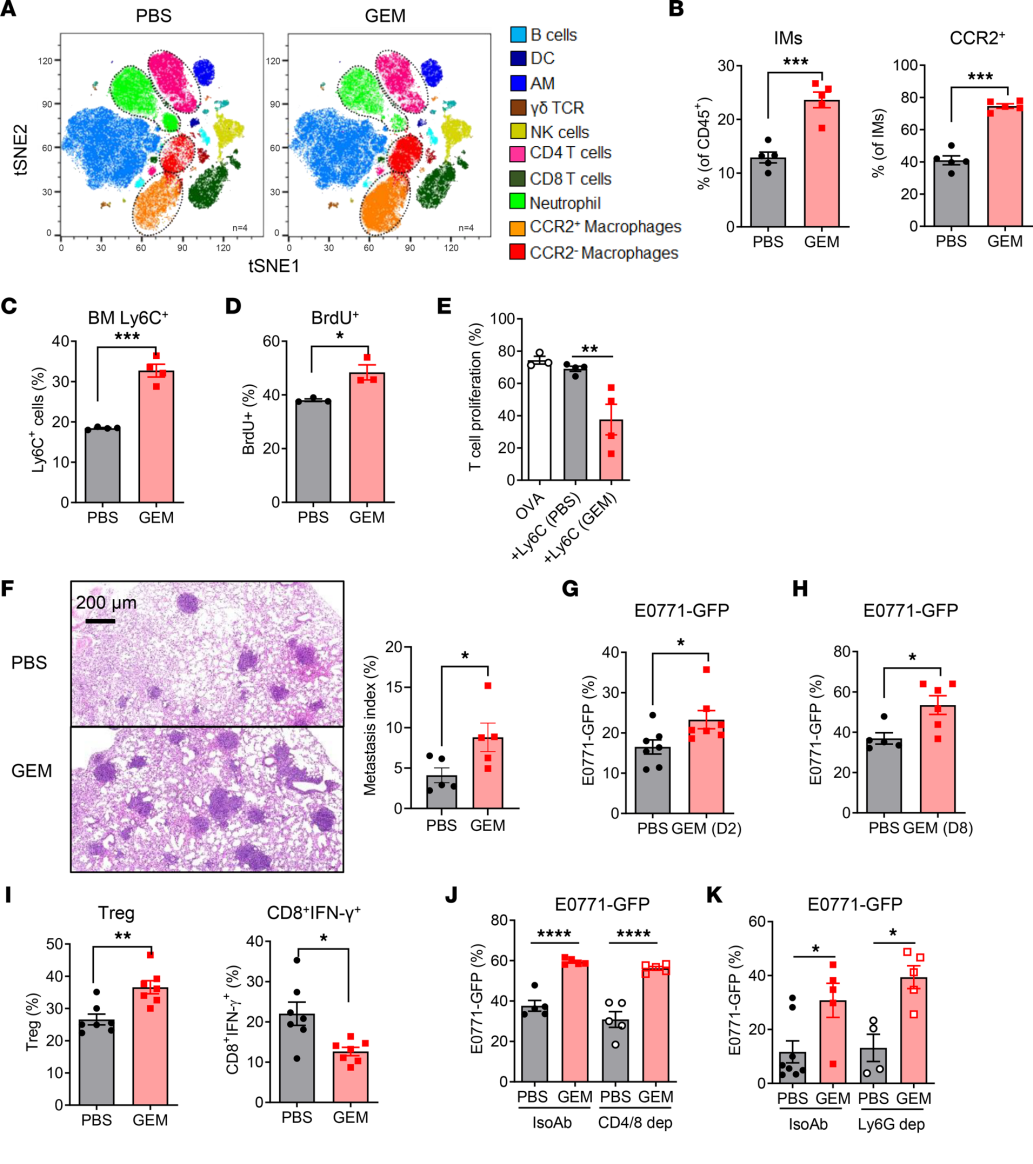
Chemotherapy-triggered host responses promote tumor metastasis in mice. Naive tumor-free C57BL/6 mice were treated with 4 doses of GEM (60 mg/kg, IP). Lung tissues and BM were harvested 2 days later after last treatment. (**A**) tSNE plot of major immune cell subsets in the lungs of PBS- and GEM-treated mice identified by FlowSOM clustering algorithm (gated on CD45^+^). (**B**) Summarized data of lung macrophages (CD11b^hi^F4/80^+^CD11c^−^) and CCR2^+^ macrophages from PBS- and GEM-treated mice (*n* = 5). (**C**) Summarized data of BM Ly6C^+^ monocytes gated on CD11b^+^ cell population (*n* = 4). (**D**) The PBS- and GEM-treated mice were IP injected with BrdU (2 mg per mouse). BM was collected 16 hours later, and the proliferating Ly6C^+^ cells stained for incorporated BrdU were analyzed by intracellular staining and flow cytometry (*n* = 3). (**E**) BM Ly6C^+^ cells from PBS- and GEM-treated mice were sorted and cocultured with CFSE-labeled OT-I splenocytes (1:1 ratio) in the presence of OVA (20 μg/mL) for 3 days. T cell proliferation was measured by flow cytometry (*n* = 4). (**F**) Tumor-free mice were treated with 4 doses of GEM and PBS, followed by intravenous injection of E0771-GFP cells (4 × 10^5^ per mouse) 2 days later after last GEM treatment. Lung metastasis was determined at day 14 by measuring metastasis index (percentages of metastasis area to lung area). (**G**) Tumor burden in lungs of GEM-pretreated mice was determined by measuring GFP^+^ tumor cells within CD45^−^ cell population (*n* = 7). (**H**) E0771-GFP cells (4 × 10^5^ per mouse) were IV injected into GEM-pretreated mice 8 days later after last GEM treatment. Lung metastasis was determined by measuring GFP^+^ tumor cells within CD45^−^ cell population (*n* = 5–6). (**I**) Regulatory T cells and IFN-γ–producing CD8^+^ T cells in GEM-pretreated tumor-bearing mice were evaluated by intracellular staining and flow cytometry (*n* = 7). (**J** and **K**) B6 tumor-free mice were treated by 4 doses of GEM and PBS. The CD4^+^/CD8^+^ depletion Abs (250 μg, IP, weekly) (**J**) or Ly6G depletion Ab (300 μg, IP, twice a week) (**K**) and IsoAb were used during the GEM or PBS pretreatment. Lung metastasis was determined by measuring GFP^+^ tumor cells within CD45^−^ cell population after IV injection of E0771-GFP cells (*n* = 5–8). Data are representative of 2 independent experiments and presented as mean ± SEM. Each dot represents 1 mouse. *P < 0.05, **P < 0.01, ***P < 0.001, and ****P < 0.0001 by ordinary 1-way ANOVA (**E**, **J**, and **K**) or unpaired 2-sided *t* test (**B**–**D** and **F**–**I**).

The roles of tumor cell–derived cytokines, chemokines, and exosomes in chemotherapy-induced metastasis have been previously investigated (13, 14). To examine whether host responses following chemotherapy also modulate lung myeloid cells, tumor-free mice were treated with GEM or PBS. Similar to the tumor-bearing mice, GEM treatment induced accumulation of macrophages (CCR2^+^CD11b^+^F4/80^+^) and monocytes (CCR2^+^Ly6C^+^) in the lungs (Figure 2, A and B). CCR2^+^ macrophages are replenished through monocyte recruitment. Thus, we further examined monocytes in the BM and found that more monocytes were generated in the BM of GEM-treated mice (Figure 2C). A BrdU incorporation assay revealed enhanced proliferation of Ly6C^+^ cells in GEM-treated mice (Figure 2D), as BrdU is only incorporated into newly synthesized DNA of proliferating cells. More importantly, BM monocytes from GEM-treated mice displayed immunosuppressive features when cocultured with OVA TCR-Tg T cells (Figure 2E). These data are consistent with a previous report that M-MDSCs have a higher proliferation rate in the BM (23).

To investigate the consequence of host-specific responses following chemotherapy on metastasis, naive mice were treated 4 times with GEM or PBS in 2 weeks, followed by intravenous injection of E0771-GFP cells. Lung metastasis was significantly higher in the GEM-pretreated mice compared with that in PBS-treated control mice, as determined by histopathological analysis with routine H&E staining (Figure 2F) and flow cytometric analysis of GFP^+^ tumor cells (Figure 2G). We performed experimentation by injection of tumor cells 8 days after chemotherapy, when BM hematopoietic stem and progenitor cells (HSPCs) recover from chemotherapy-induced stress (24). Lung metastasis was also higher in the GEM-pretreated mice compared with that in PBS-treated control mice (Figure 2H). To determine whether antitumor immunity is modulated in the GEM-pretreated mice, the phenotype of T cells and NK cells was evaluated in the PBS- or GEM-pretreated E0771 tumor–bearing lungs. The effector T cells (CD8^+^IFN-γ^+^) were decreased, whereas Tregs were significantly increased in the GEM-pretreated mice (Figure 2I). The total numbers of CD45^+^ cells and Tregs were also increased in the GEM-pretreated mice, causing a decrease of the ratios of effector T cells and NK cells to Tregs in the GEM-pretreated mice (Supplemental Figure 2). These data support the importance of host responses in the chemotherapy-induced lung metastasis.

Chemotherapy has been shown to modulate T cell compartment and function in patients with breast cancer (25). Previous studies also revealed that Ly6G^+^ neutrophils support lung colonization of metastatic cancer cells (26). To test whether T cells and neutrophils are critical for the pro-metastatic effect triggered by chemotherapy, anti-CD4 and -CD8, as well as anti-Ly6G, depletion antibodies were used during the GEM or PBS treatment period. The depletion efficiency of CD4^+^, CD8^+^, and Ly6G^+^ cells is shown in Supplemental Figure 3, A and B. No difference of lung tumor burdens was observed in the IsoAb- and T cell–depleted or neutrophil depleted GEM-pretreated mice (Figure 2, J and K). These data demonstrate the importance of monocytes and BM-derived macrophages, but not T cells and neutrophils, in pro-metastatic effects of host response triggered by chemotherapy.

### GEM treatment induces reactive myelopoiesis with enhanced myeloid potential.

**Figure 3:**
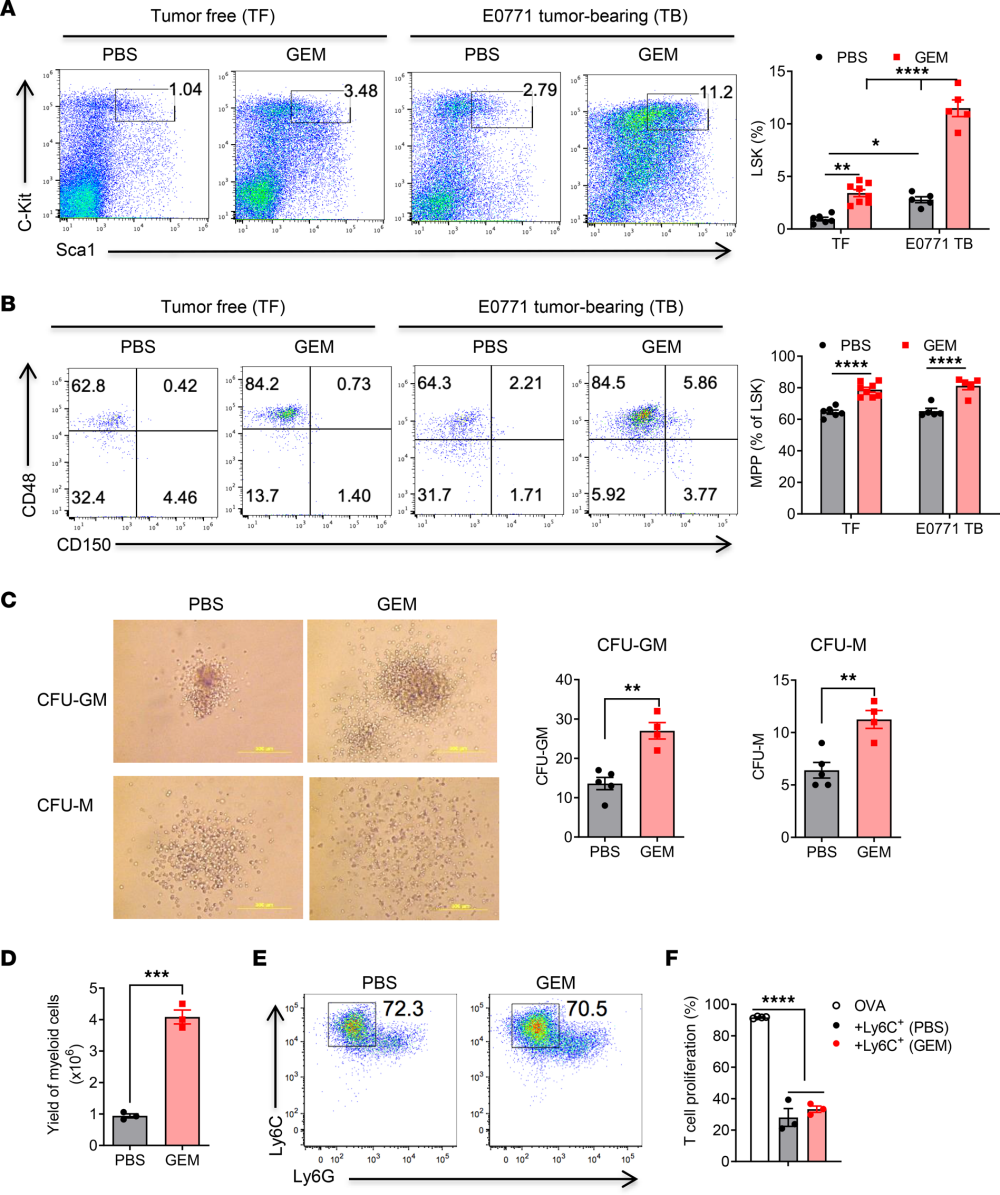
GEM treatment induces reactive myelopoiesis in tumor-free and tumor-bearing mice with enhanced myeloid potential. Naive C57BL/6 and E0771 tumor–bearing mice were treated with 4 doses of GEM (60 mg/kg, IP). BM was harvested 2 days later after last treatment. (**A**) Representative FACS plots gated on lineage-negative cells and summarized data of LSK cells (Lin^–^Sca1^+^c-Kit^+^) (*n* = 5–8). (**B**) Representative FACS plots gated on LSK cells and summarized data of MPPs (CD150^–^CD48^+^) (*n* = 5–8). (**C**) Representative CFU-GM and CFU-M after 7 days’ culture of BM cells from GEM-treated tumor-free mice in MethoCult GF M3534 methylcellulose-based medium. Numbers of CFU-GM and CFU-M were summarized (*n* = 4–5). Scale bar, 500 μm. (**D**) BM cells (1 × 10^6^) were cultured in the presence of 20% E0771-conditioned medium (CM) for 2 days. The culture medium including nonadherent cells was entirely discarded at day 3 and replaced by medium containing E0771 CM for an additional 4 days. The yield of myeloid cells was counted after 6 days’ culture (*n* = 3). (**E**) Representative FACS plots of Ly6G^–^Ly6C^+^ in vitro–expanded cells. (**F**) Sorted Ly6G^–^Ly6C^+^ cells were cultured with CFSE-labeled OT-I splenocytes in the presence of OVA (20 μg/mL) for 3 days. T cell proliferation was measured by flow cytometry (*n* = 3). Data are representative of 2 or 3 independent experiments and presented as mean ± SEM. *P < 0.05, **P < 0.01, ***P < 0.001, and ****P < 0.0001 by ordinary 1-way ANOVA (**A**, **B**, and **F**) or unpaired 2-sided *t* test (**C** and **D**).

Lung macrophages include tissue-resident macrophages and BM-derived macrophages, which are differentiated from CCR2^+^ classical monocytes. We hypothesized that the elevated frequency of monocytes/macrophages after GEM treatment may arise from an increased number of BM myeloid progenitor cells that generate more BM monocytes. To determine whether GEM treatment induces reactive myelopoiesis, tumor-free and E0771 tumor–bearing mice were treated 4 times with GEM or PBS. E0771 primary tumor development induced an increase of BM Lin^−^Sca1^+^c-Kit^+^ (LSK) cells and multipotent progenitors (MPPs, CD48^+^CD150^−^). GEM treatment further increased the accumulation of BM LSK cells and MPPs (Figure 3, A and B).

To examine the ability of BM progenitors to differentiate into monocytes and macrophages, we performed a colony formation assay using BM cells from GEM- or PBS-treated mice. After 7 days of culture, both colony numbers of granulocyte/monocyte colony-forming units (CFU-GM) and macrophage colony-forming units (CFU-M) from GEM-treated BM cells were increased as compared with that from PBS-treated BM cells (Figure 3C). No changes of granulocyte colony-forming units were observed between the 2 groups. We further cultured BM cells from GEM- or PBS-treated mice in the presence of E0771 tumor cell–conditioned medium (CM). There was a significant increase in the yield of cells from GEM-treated BM cells after 6 days’ in vitro culture (Figure 3D). The major cell population displayed the phenotype of monocytes (CD11b^+^Ly6C^+^Ly6G^−^) (Figure 3E). Importantly, these in vitro–differentiated cells exhibited potent immunosuppressive function when cocultured with T cells (Figure 3F). Together, these data suggest that multidose chemotherapy may induce reactive myelopoiesis with myelopoietic bias that boosts monocyte development and expansion, ultimately leading to the accumulation of immunosuppressive monocyte-derived macrophages in the lungs.

### Upregulation of mitochondrial ROS in the BM microenvironment triggered by GEM treatment.

**Figure 4:**
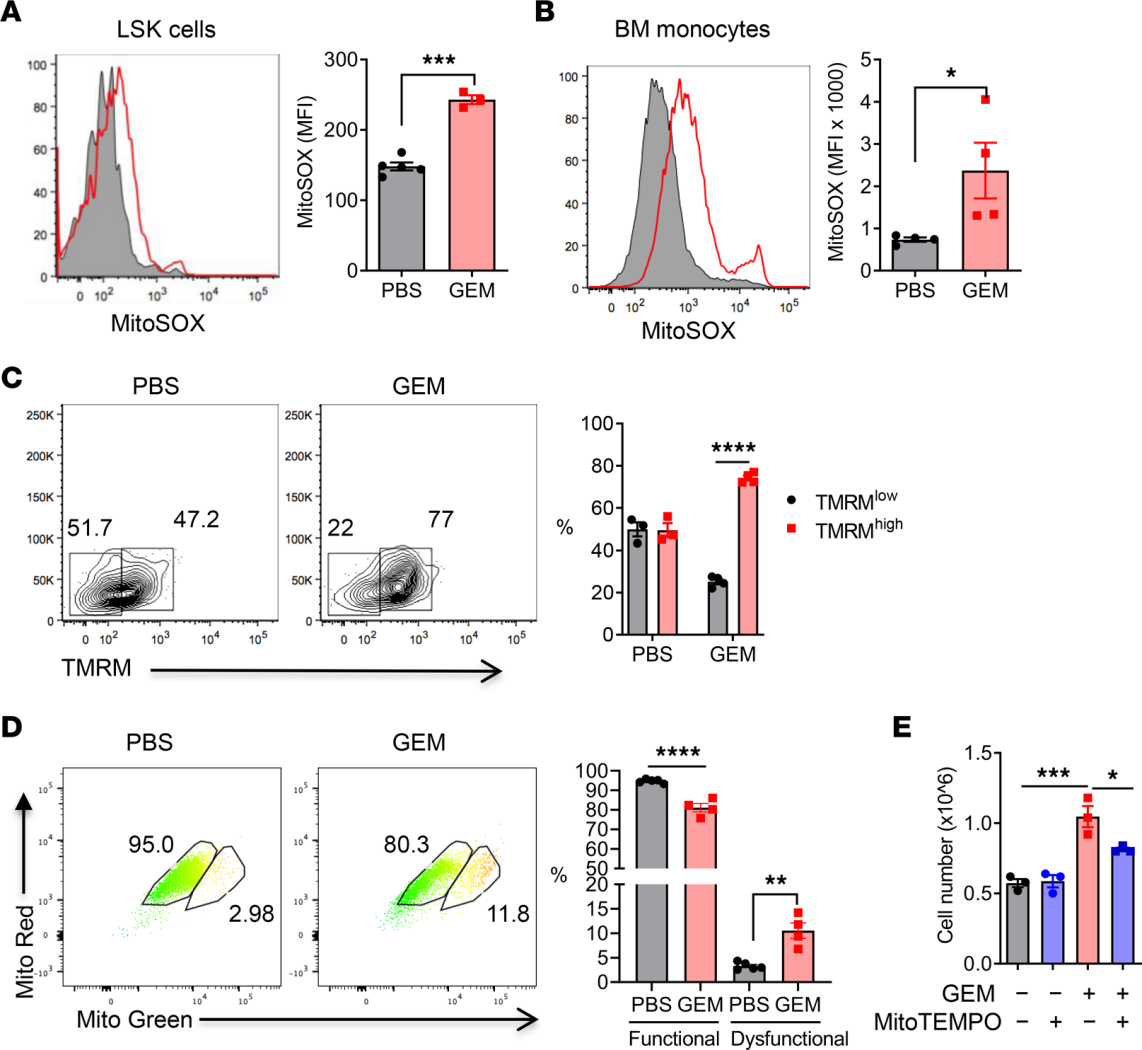
Upregulation of mtROS in BM microenvironment triggered by GEM treatment. Naive tumor-free C57BL/6 mice were treated with 4 doses of GEM (60 mg/kg, IP). BM was harvested 2 days later after the last treatment. (**A** and **B**) mtROS production in LSK cells (**A**) and BM monocytes (**B**) was determined by using MitoSOX dye (5 μM) (*n* = 3–5). (**C**) Mitochondria potential was determined by using TMRM dye (500 nM) followed by lineage marker and Sca1 and c-Kit antibody staining. The percentages of TMRM^hi^ and TMRM^lo^ LSK cells were summarized (*n* = 3–4). (**D**) BM cells were stained with MitoTracker Green and MitoTracker Red followed by BM LSK cell markers. Representative plots gated on LSK cells and summarized functional and dysfunctional mitochondria were shown (*n* = 4). (**E**) BM cells (1 × 10^6^) from PBS- and GEM-pretreated mice were treated with MitoTEMPO (10 μM) and cultured in the presence of GM-CSF (20 ng/mL) for 2 days. The culture medium including nonadherent cells was entirely discarded at day 3 and replaced by medium containing GM-CSF and MitoTEMPO for an additional 4 days. Cell numbers of CD11b^+^Ly6C^+^ monocytes after 6 days’ culture was determined by cell counting and flow cytometry (*n* = 3). Data are representative of 2 experiments and presented as mean ± SEM. *P < 0.05, **P < 0.01, ***P < 0.001, and ****P < 0.0001 by ordinary 1-way ANOVA (**C**–**E**) or unpaired 2-sided *t* test (**A** and **B**).

In response to various types of inflammation, HSPCs undergo a metabolic switch from glycolysis to oxidative phosphorylation (OXPHOS) that leads to increased ROS production, which is important for HSPC proliferation and differentiation (27–31). We observed a significant increase of mitochondrial ROS (mtROS) in LSK cells (Figure 4A) and monocytes (Figure 4B) from mice that received GEM treatment. Mitochondria that have a high membrane potential are more prone to ROS generation (32). Thus, we measured mitochondrial membrane potential by using tetramethylrhodamine, methyl ester (TMRM), staining and found higher mitochondrial potential in LSK cells of GEM-treated mice (Figure 4C). Mitochondrial dysfunction is associated with increased ROS production (33). We further stained BM cells with MitoTracker Green and MitoTracker Red to distinguish between functional mitochondria (MitoTracker Red^hi^) and dysfunctional mitochondria (MitoTracker Green^hi^, MitoTracker Red^+/lo^) (34). We observed an increase of dysfunctional mitochondria but decrease in functional mitochondria in GEM-treated LSK cells compared with those in control mice (Figure 4D). These data suggest that GEM treatment has the potential to modulate HSPC mitochondrial activity and metabolism.

Emerging studies suggest that ROS also act as signal-transducing molecules that drive HSPCs’ self-renewal and emergency granulopoiesis (35). To determine the importance of mtROS in GEM treatment–induced myelopoiesis, BM cells from GEM and control mice were treated with mitochondria targeted superoxide scavenger mitoTEMPO and then cultured in the presence of GM-CSF (36). Significant increase of in vitro–differentiated monocytes was observed from the BM of GEM-treated mice, and inhibition of mtROS using mitoTEMPO abrogated GEM treatment–induced high yield of monocytes (Figure 4E). These results suggest that mtROS production in BM niche might play an important role in chemotherapy-induced BM myelopoiesis and monocyte development.

### Chemotherapy-induced upregulation of macrophage-synthesized FX contributes to lung metastasis.

**Figure 5:**
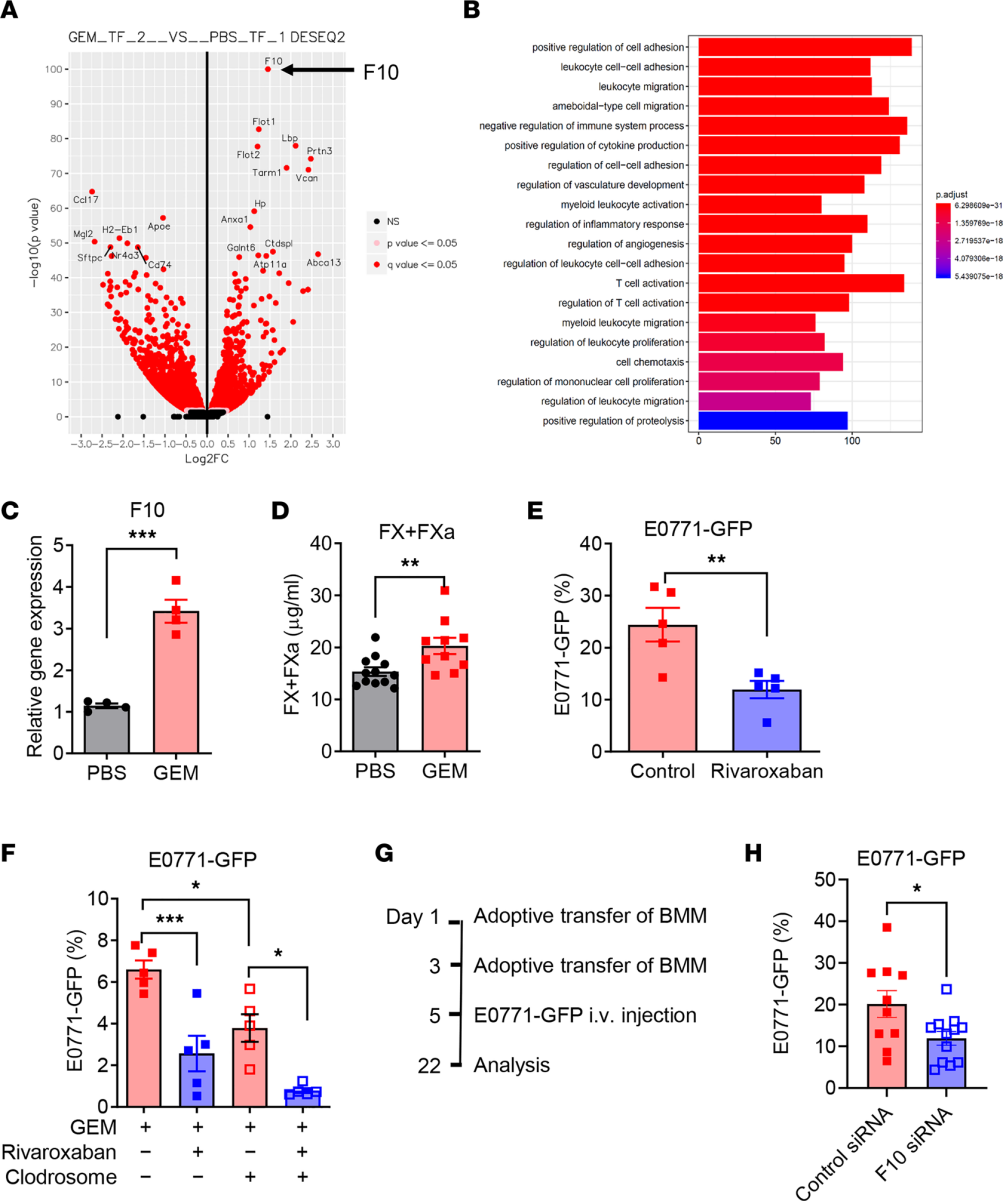
Chemotherapy-induced upregulation of macrophage-synthesized FX contributes to lung metastasis. (**A**) RNA-Seq analysis in lung macrophages sorted from naive mice after GEM and PBS treatment (*n* = 3). Volcano plot for differential gene expression in lung macrophages from GEM-treated mice as compared with PBS-treated mice. (**B**) Top 20 enriched GO biological processes for the genes in lung macrophages from GEM-treated mice as compared with PBS-treated mice. (**C**) qRT-PCR for *F10* gene in lung macrophages from GEM- and PBS-treated mice. (**D**) Levels of total FX and FXa in the plasma of GEM- and PBS-treated mice (*n* = 10–12). (**E**) Naive mice were treated with GEM and rivaroxaban, or solvent control, followed by IV injection of 4 × 10^5^ E0771-GFP cells. The lung tumor burden was examined by measuring GFP^+^ tumor cells within lung CD45^–^ cells 14 days after tumor cell injection (*n* = 5). (**F**) Mice were treated with GEM and rivaroxaban, or solvent control, followed by IV injection of 1 × 10^5^ E0771-GFP cells. Lung macrophages were depleted by IV injection of clodrosome during the GEM/rivaroxaban treatment and tumor development. The lung tumor burden was examined by measuring GFP^+^ tumor cells within lung CD45^–^ cells 14 days after tumor cell injection (*n* = 5). (**G**) Experimental scheme for adoptive transfer of E0771 CM–stimulated (24 hours) control and *F10* gene–knockdown macrophages (2 × 10^6^/mouse) and E0771-GFP (4 × 10^5^/mouse) injection. (**H**) The lung tumor burden was examined by measuring GFP^+^ tumor cells within lung CD45^–^ cells 17 days after tumor cell injection (*n* = 10–12). The data in **H** represent a combination of 2 experiments. Each dot represents 1 mouse. *P < 0.05, **P < 0.01, and ***P < 0.001 by unpaired 2-sided *t* test (**C**, **D**, **E**, and **H**) or ordinary 1-way ANOVA (**F**).

To identify factors that may contribute to the phenotypic changes of lung macrophages after GEM treatment, we performed an unbiased gene expression profiling analysis of macrophages from GEM- and PBS-treated mice. A total of 2,712 differentially expressed genes (DEGs) were recorded (1,315 upregulated DEGs and 1,397 downregulated DEGs). Gene *F10* encoding FX was identified as one of the most upregulated DEGs in the macrophages from GEM-treated mice (Figure 5A), which was validated by using quantitative real-time PCR (qRT-PCR) (Figure 5C). Gene ontology (GO) analysis revealed a pattern of enrichment in pathways related to leukocyte adhesion and migration, negative regulation immune cell process, as well as regulation of vasculature development (Figure 5B). Additionally, protein levels of total FX and FXa were significantly increased in the plasma of mice that received GEM treatment (Figure 5D). Further, several pro-metastatic molecules, including *S100A8*, *S100A9*, and *TGFB1*, were upregulated in the lung tissues after GEM treatment (Supplemental Figure 4).

To determine the role of macrophage-expressed FX in the GEM-induced pro-metastatic effect in vivo, mice were pretreated with GEM along with rivaroxaban, an oral inhibitor of FXa, and vehicle control daily throughout the GEM pretreatment period. E0771-GFP cells were intravenously injected into mice 2 days later after the last GEM treatment. As shown in Figure 5E, inhibition of FXa using FXa inhibitor rivaroxaban significantly reduced the pro-metastatic effect of host response triggered by GEM treatment. To address whether the antimetastatic effect of FXa inhibitor is mediated by macrophage-expressed FX, we depleted lung macrophages by intravenous injection of clodrosome. The experimental scheme for in vivo GEM and rivaroxaban treatment and macrophage depletion efficacy is shown in Supplemental Figure 3, C and D. Depletion of macrophages resulted in reduced tumor burden in the GEM-pretreated mice. The antimetastatic effect of FXa inhibition was also observed in the macrophage-depleted mice (Figure 5F). These data suggest that lung macrophages are critical for the pro-metastatic effect of chemotherapy. The off-target effect of FXa inhibition may exist.

To further determine the roles of macrophage-specific FX in the pro-metastatic effect of chemotherapy, we performed in vitro *F10* gene knockdown in BM-derived macrophages using *F10* siRNA. The knockdown efficiency is shown in Supplemental Figure 5. The E0771 CM–treated *F10*-knockdown and control macrophages were adoptively transferred into CCR2-KO recipient mice twice, 48 hours apart, followed by intravenous injection of E0771-GFP cells (Figure 5G). As shown in Figure 5H, the mice transferred with *F10*-knockdown macrophages had a lower percentage of GFP^+^ tumor cells compared with mice transferred with control macrophages, indicating the contribution of macrophage-expressed FX in pro-metastatic effects of chemotherapy.

Intravenous cancer cell injection does not recapitulate all the steps of metastasis. We performed experiments to further evaluate the antimetastatic effect of rivaroxaban in the E0771-GFP subcutaneous tumor model. E0771 is poorly metastatic as compared with 4T1 tumors. We performed 3 rounds of in vivo passages. E0771-GFP cells recovered from tumor-bearing lungs can develop pulmonary metastases. Mice bearing E0771-GFP subcutaneous tumors were treated with rivaroxaban (oral, 20 mg/kg, daily) or solvent control for 2 weeks. As shown in Supplemental Figure 6, rivaroxaban treatment significantly decreased lung metastasis, which was observed in 3 out of 14 rivaroxaban-treated mice compared with 9 out of 14 control mice. The mechanism underlying the antimetastatic effect of FXa inhibitor in primary tumor–bearing mice needs to be further defined.

### GEM-induced pro-metastatic host response is dependent on CCL2/CCR2-mediated recruitment of monocytes.

**Figure 6:**
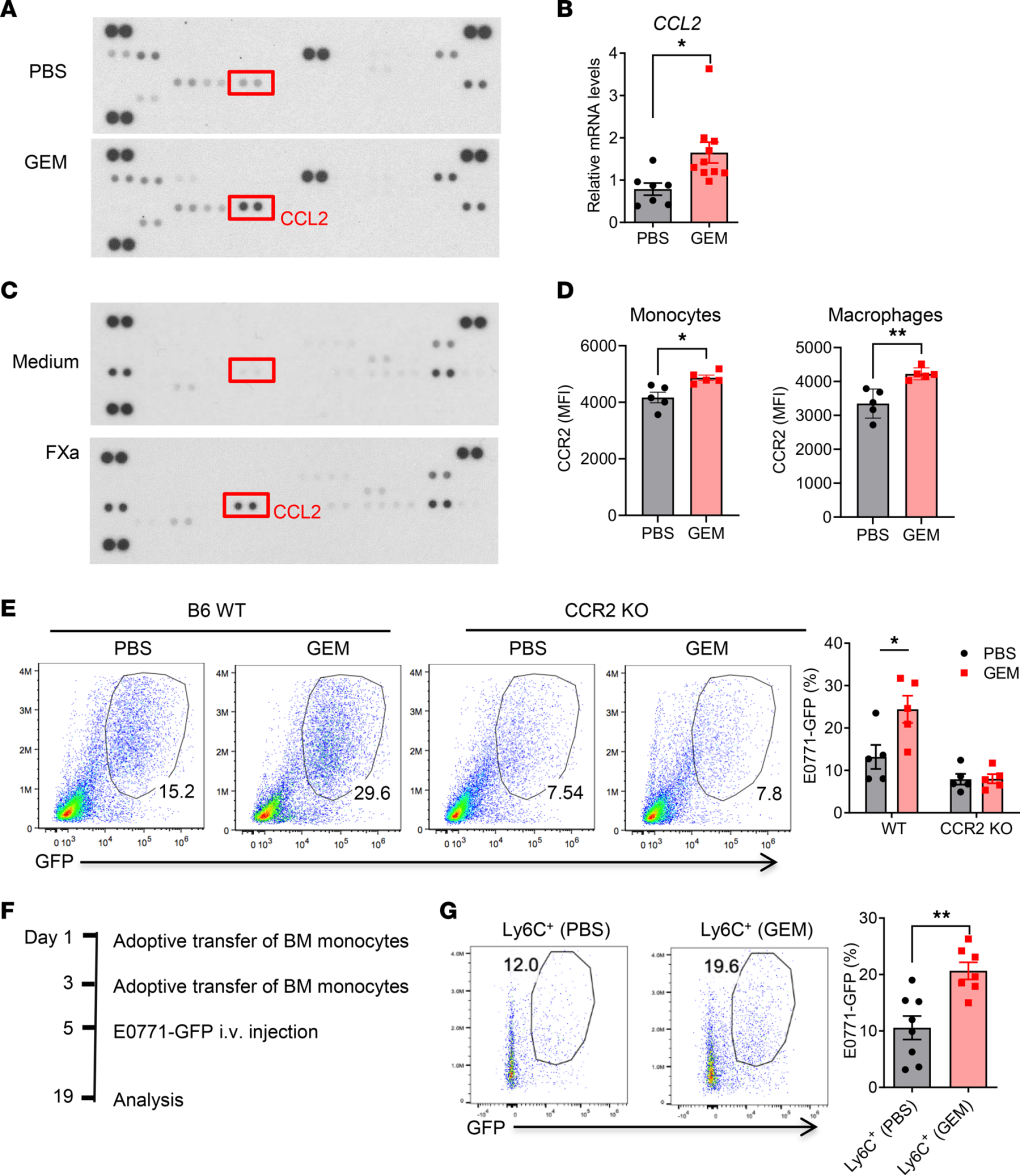
GEM-induced pro-metastatic host response is dependent on CCL2/CCR2-mediated recruitment of monocytes. (**A**) Mouse cytokine/chemokine array of pooled plasma from the PBS- or GEM-treated mice. (**B**) qRT-PCR for CCL2 gene in lung tissues from GEM- and PBS-treated mice (*n* = 7–10). (**C**) Lung macrophages were sorted from naive mice and stimulated with recombinant mouse FXa (Haematologic Technologies, 1 U/mL) for 24 hours. The mouse cytokine/chemokine profile in the culture supernatant was examined using cytokine/chemokine array. (**D**) Mean fluorescence intensity of CCR2 expression on lung monocytes and macrophages (*n* = 5). (**E**) B6 WT and CCR2-KO mice were treated with 4 doses of GEM followed by intravenous injection of 4 × 10^5^ E0771-GFP cells. The percentage of GFP^+^ tumor cells within CD45^–^ cell population was summarized (*n* = 5). (**F**) Experimental scheme for adoptive transfer of BM monocytes (1.5 × 10^6^/mouse) from PBS- and GEM-treated mice and E0771-GFP (4 × 10^5^/mouse) injection. (**G**) The lung tumor burden was examined by measuring GFP^+^ tumor cells within lung CD45^–^ cells 14 days after tumor cell injection (*n* = 7–8). *P < 0.05 and **P < 0.01 by unpaired 2-sided *t* test (**B**, **D**, and **G**) or ordinary 1-way ANOVA (**E**).

Primary tumor–derived CCL2 can enhance breast cancer metastasis assisted by the recruitment of Ly6C^+^CCR2^+^ monocytes (37) and retention of MAMs (38). To understand the roles of soluble factors in chemotherapy-triggered host responses, plasma was collected from GEM-treated and control mice. The cytokine/chemokine array revealed that CCL2 is the most highly expressed chemokine in the plasma of GEM-treated tumor-free mice (Figure 6A). qRT-PCR analysis also showed that *CCL2* mRNA expression was increased in the lung tissues of GEM-treated mice (Figure 6B). Previous studies have demonstrated that FXa is able to induce proinflammatory responses in cardiac fibroblasts (39). To assess whether hyperexpression of FXa has an immune modulatory effect on lung macrophages, we purified lung macrophages from naive mice and stimulated the cells with recombinant mouse FXa. Cytokine/chemokine array revealed that the level of CCL2 was significantly increased in the culture supernatants of FXa-stimulated cells (Figure 6C). Further, CCR2 expression on lung monocytes and macrophages was also increased after GEM treatment (Figure 6D). Next, we used CCR2-KO mice to determine the importance of the CCL2/CCR2 axis in the GEM-induced pro-metastatic effect. As shown in Figure 6E, deficiency of CCR2 signaling completely abrogated the pro-metastatic effect of GEM treatment.

Functional diversity of monocytes is well recognized because their development can be altered in respond to stimuli (40–43). Our RNA-Seq data of lung macrophages from GEM- versus PBS-treated mice also revealed that proteinase 3, a serine protease enzyme expressed mainly in neutrophils, was highly expressed in lung macrophages from GEM-treated mice (Supplemental Figure 7A). Lung macrophages from GEM-treated mice also demonstrated enrichment of gene signature of neutrophils (Supplemental Figure 7B). We further tested whether lung monocytes from these mice express other neutrophil signature molecules. Indeed, neutrophil elastase and *S100A8* were increased in lung monocytes from GEM-treated mice (Supplemental Figure 7C). These data suggest that chemotherapy alone induces phenotypic changes of lung monocytes. To further address the roles of BM monocytes generated via reactive myelopoiesis, BM monocytes were purified from PBS- and GEM-treated B6 WT mice and adoptively transferred into CCR2-KO mice, which are defective in monocyte egress from the BM (Figure 6F). Transferred BM monocytes could differentiate into CD11b^+^F4/80^+^ macrophages in lungs (Supplemental Figure 8). We further challenged these mice by intravenous injection of E0771-GFP cells. As shown in Figure 6G, higher tumor burden was observed in the mice transferred with GEM-treated BM monocytes compared with the mice transferred with control monocytes. These data suggest that reactive myelopoiesis and the CCL2/CCR2 axis play essential roles in GEM-induced recruitment of monocytes and macrophages into the lungs, which ultimately contribute to the chemotherapy-induced pro-metastatic effect.

### PTX and DOX treatment elicits host responses like GEM.

**Figure 7:**
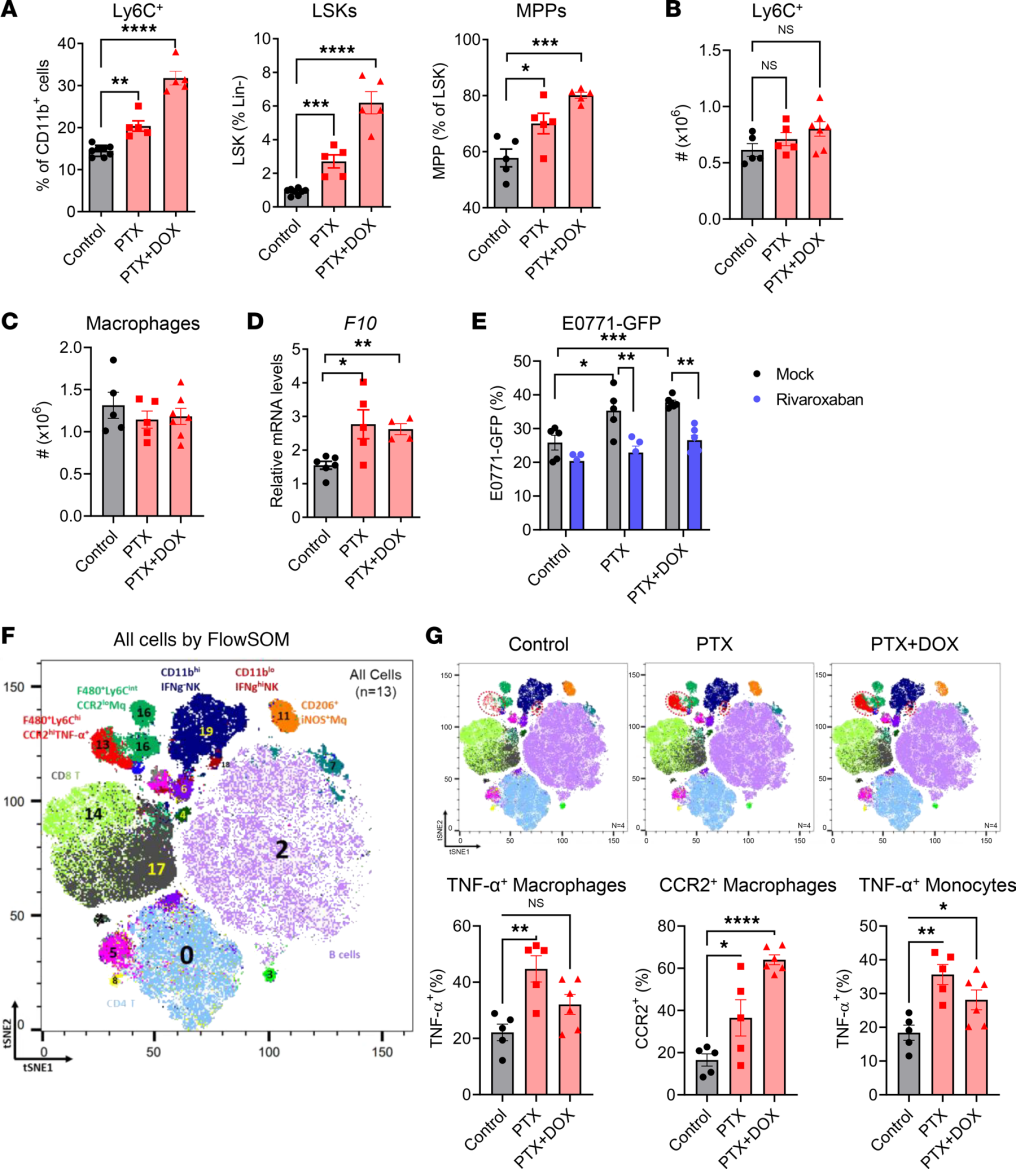
PTX or combination of PTX and DOX treatment elicits host responses like GEM. B6 WT mice were treated PTX (10 mg/kg, IP) or combination of PTX and DOX (2 mg/kg) or Cremophor EL control 3 times in 2 weeks. Lungs and BM were collected 2 days later after last treatment. (**A**) Percentages of BM Ly6C^+^ monocytes, LSK cells, and MPPs in BM of treated and control mice (*n* = 5). (**B**) Total cell numbers of lung Ly6C^+^ monocytes (*n* = 5–7). (**C**) Total cell numbers of lung macrophages (CD11b^hi^F4/80^+^CD11c^−^) (*n* = 5–7). (**D**) qRT-PCR for *F10* gene in lung macrophages from treated and control mice (*n* = 4–5). (**E**) B6 WT mice were treated with PTX or combination of PTX and DOX or Cremophor EL control along with rivaroxaban or solvent control (mock) for 2 weeks. E0771-GFP cells (4 × 10^5^) were intravenously injected into these mice 2 days later after last treatment. The percentage of GFP^+^ tumor cells in the lung tissues was determined 2 weeks later (*n* = 5–6). (**F** and **G**) Lung cells were stimulated with PMA/ionomycin (MilliporeSigma) in the presence of Brefeldin A (BioLegend) for 4–6 hours, then permeabilized for surface and intracellular staining. tSNE analysis of CyTOF immunophenotyping of the lungs from mice treated with PTX, PTX plus DOX, or solvent control (*n* = 4–5). All samples combined (**F**), combined samples from each group (**G**, top), and frequencies in different groups (**G**, bottom). Cell population 13 (red) is TNF-α–producing CCR2^+^ macrophages. Data are representative of 2 independent experiments and presented as mean ± SEM. *P < 0.05, **P < 0.01, ***P < 0.001, and ****P < 0.0001 by ordinary 1-way ANOVA (**A**, **B**, **D**, **E**, and **G**).

GEM is used in patients with locally advanced or metastatic breast cancer. Having shown that GEM treatment induces pro-metastatic host responses, we next examined whether similar effects could be triggered by other chemotherapeutic drugs. PTX and DOX are commonly used for the treatment of human breast cancer. Tumor-free mice received a total of 3 doses of PTX alone or the combination of PTX and DOX and solvent control in 2 weeks. As shown in Figure 7, PTX and the combination of PTX and DOX treatment induced similar BM myelopoiesis to GEM treatment, including increased BM monocytes, LSK cells, and MPPs (Figure 7A). Although total cell numbers of lung monocytes and macrophages were not significantly increased (Figure 7, B and C), higher expression of FX was observed in the lung macrophages after PTX or PTX plus DOX treatment (Figure 7D). Importantly, pretreatment with PTX or the combination of PTX and DOX also induced a pro-metastatic effect as revealed by increased lung metastasis in PTX- and PTX plus DOX–pretreated mice. Inhibition of FX using FXa inhibitor rivaroxaban significantly reduced PTX- and PTX plus DOX–induced pro-metastatic effects (Figure 7E).

To profile lung immune cells and determine their changes in response to chemotherapy, we used flow cytometry and CyTOF to assess immune cells from control and PTX- or PTX plus DOX–treated tumor-free mice. The total cell numbers of effector CD4^+^, CD8^+^ T cells, and NK cells, as well as immunosuppressive Tregs, were not substantially changed in the chemotherapy-treated tumor-free mice (Supplemental Figure 9). However, CyTOF analysis identified a significant expansion of TNF-α–producing CCR2^+^ macrophages and monocytes (population 13, red) in the lungs of chemotherapy-treated mice (Figure 7, F and G), indicating that chemotherapy preconditioning can induce a hyperinflammatory state, which is one of the characteristics of PMN formation (44–46). Taken together, these findings further support the link between chemotherapy-induced monocyte/macrophage expansion and recruitment, FX expression by these myeloid cells, and FX-induced pro-metastatic effect.

## Discussion

About 90% of cancer-related deaths are due to metastasis, and yet currently there are no broadly effective ways to prevent or cure metastatic cancers (47). Chemotherapy is a mainstay of cancer treatment, but in addition to its benefits, it can have the undesired effect of promoting immunosuppression and metastasis (11–13, 48). Previous studies in this area have focused mainly on tumor cell–derived cytokines, chemokines, and exosomes. A recent study showed that chemotherapy elicits pro-metastatic tumor-secreted extracellular vesicles in breast cancer models (14). We also demonstrate that multidose chemotherapy enhances the accumulation of immunosuppressive Ly6C^+^ cells in primary tumor microenvironment via the activation of GM-CSF in tumor cells (18). The current study demonstrates a potentially previously unidentified mechanism by which host responses characterized by accumulation of monocytes/macrophages and upregulation of macrophage-expressed FX contribute to chemotherapy-induced metastasis promotion. This likely previously unrecognized mechanism of chemotherapy-induced metastasis is not restricted to GEM, because we found that PTX and DOX treatment has similar effects.

Lung metastasis is the lethal determinant in many cancers. Normal lung tissues constitutively contain abundant myeloid cells, which include monocytes, alveolar macrophages, interstitial macrophages (IMs), and neutrophils (4, 7, 49–51). IMs markedly accumulate and differentiate into MAMs in metastatic lung cancer (4). Regarding the origin, lung IMs/MAMs include embryonic tissue-resident macrophages and CCR2-dependent recruited macrophages. Systemic inflammation of primary tumor drives CCR2^+^Ly6C^+^ monocytes’ migration into lung PMN and differentiation into MAMs (38). Our recent studies have demonstrated that lung IMs are responsible for tumor-derived exosome-induced immunosuppression in the PMN (52), and inducing trained immunity in IMs leads to antimetastatic activities (53). The current study further demonstrates that BM-derived lung IMs/MAMs are the primary effector cells that mediate the pro-metastatic effect of chemotherapy. This conclusion is supported by findings that (a) the host response triggered by chemotherapy promoted monocyte accumulation in the lungs and differentiation into macrophages in the presence or absence of primary tumors; (2) in vivo macrophage, neutrophil, and CD4^+^/CD8^+^ T cell depletion studies demonstrated that the pro-metastatic effect of chemotherapy was dependent on macrophages but not on neutrophils and T cells; (3) the pro-metastatic effect of chemotherapy was abrogated in CCR2-KO mice; and (4) adoptive transfer of BM monocytes into CCR2-KO mice promoted lung metastasis after intravenous injection of tumor cells.

BM suppression is a common side effect of chemotherapy. We observed pro-metastatic effects of chemotherapy not only at myelosuppression but also at BM recovery phase. Although onetime treatment with chemotherapy results in a decrease of MDSCs (54, 55), cell depletion induced by cytotoxic drugs might increase myelopoiesis rates to meet the need to replenish depleted reserves (27, 28). This phenomenon is known as reactive myelopoiesis (56). The majority of mobilized HSPCs then differentiate into CD11b^+^Gr-1^+^ cells, which limits the efficacy of adoptive T cell therapy (57). In this study, we showed that GEM, PTX, or PTX plus DOX treatment resulted in myelopoiesis leading to enhanced development of monocytes but not neutrophils. The difference in myeloid cell development might be due to the different progenitor cells (58, 59). For example, in response to CpG-DNA, monocyte-DC progenitors (MDPs) yielded monocytes and dendritic cells. In contrast, administration of LPS produced neutrophils and monocytes directly from granulocyte-monocyte progenitors (GMPs) (59). Further, tumor cells with high metastatic potential enriched MDPs that functionally differentiated into immunosuppressive monocytes to support the metastatic switch (58). Our future study will investigate the effect of chemotherapy treatment on the GMP and MDP. Cytokines and chemokines play important roles in regulating myelopoiesis and HSPC fate decision. In addition to cytokines, ROS also play important roles in regulating HSPCs’ proliferation and differentiation. In response to inflammation, HSPCs require a metabolic switch from glycolysis to mitochondrial OXPHOS leading an increase of ROS production (60). We showed that GEM treatment increased mtROS production by LSK cells and BM monocytes. These data led us to hypothesize that mtROS might be responsible for GEM-induced monocyte development. Indeed, inhibition of mtROS using mitochondria targeted superoxide scavenger mitoTEMPO abrogated GEM-induced hyperdifferentiation of BM progenitors.

Several molecules, such as VEGFR1 (6), MMP1 (9), TGF-β (61), and IL-35 (62), have been identified to participate in the pro-metastatic effects of lung macrophages. However, current strategies targeting macrophage-associated molecules have shown limited success in clinical settings (3, 63). Cancer progression results in a higher risk of thromboembolism in patients (64, 65). Recent studies demonstrated that some coagulation factors play important roles in tumor progression and metastasis (64–68). For example, factor XIIIA expressed on lung inflammatory monocytes promoted fibrin cross-linking to create a scaffold for lung squamous cancer cell invasion and metastases (69). FXa treatment promoted tumor and metastasis by inducing endothelial cell activation (66). Additionally, FX expression in myeloid cells within tumor microenvironments resulted in upregulation of programmed cell death ligand 1 and impaired antitumor immunity (70). Some chemotherapy drugs, such as GEM and cisplatin, have been associated with an increased risk of thrombotic events (71). A study using factor VIIa inhibitor in combination with GEM showed a nonsignificant trend toward longer progression-free survival in patients with cancer (72). Our study identified *F10* as a highly expressed DEG in the lung macrophages of tumor-free mice after chemotherapy, suggesting that non–tumor cell–derived factors can induce a hypercoagulable state. Inhibition of FX using oral available FXa inhibitor rivaroxaban or *F10* gene–knockdown in macrophages substantially decreased the chemotherapy-induced pro-metastatic effect. Previous studies have shown that locally expressed FX contributed to the fibrotic response in bleomycin-induced lung injury (73). Thus, the fibrotic reactions and remodeling of extracellular matrix in lungs might be a potential mechanism of chemotherapy-induced metastasis. FX occupies a central position in the coagulation cascade, as it can be activated via both the intrinsic and extrinsic pathways (67, 74); our findings also suggest that FXa might be a better target for potential combination therapy of chemotherapy and anticoagulation.

A limitation of this study is using the GEM-nonresponsive MMTV-PyMT tumor model. Our previous studies have shown that GEM treatment significantly delays the primary tumor progression in E0771 subcutaneous tumor (18). The current study did not observe a similar effect in MMTV-PyMT mice. This might be due to the phenotypic difference of the tumor models (21, 22). GEM-nonresponsive MMTV-PyMT primary tumor progression also could be a net effect of the cytotoxic effect of chemotherapy drugs and the pro-tumor effect of reactive myelopoiesis–induced systemic immunosuppressive effect. There are 2 reasons we used MMTV-PyMT model: (a) E0771 is poorly metastatic (75), and (b) it is not comparable to examine the pro-metastatic effect of chemotherapy if the primary tumor sizes are different in chemotherapy-treated and control mice. We understand that use of a chemotherapy-nonresponsive tumor model has limited clinical relevance, but it provides a model to demonstrate the pro-metastatic effect of chemotherapy via the chemotherapy-induced changes in host responses as well as tumor microenvironment.

## Methods

### Mice, tumor models, and chemotherapy in vivo treatment.

C57BL/6J mice, CCR2-KO mice, and Tg FVB/MMTV-PyMT mice were purchased from The Jackson Laboratory. Rag2-deficient OVA TCR-Tg OT-I mice were purchased from Taconic Biosciences. When maintaining a live colony of FVB/MMTV-PyMT mice, FVB/NJ inbred females were bred with hemizygous males. The pups were genotyped by PCR. Only female Tg mice were used in studies. All animals were maintained under specific pathogen–free conditions. The murine mammary cancer cell lines E0771 (from CH3 BioSystems) and E0771-GFP cells (Yan Lab, UofL Health - Brown Cancer Center) were confirmed pathogen free by VRL Diagnostics without further authentication. Cells were cultured in complete DMEM containing 10% FBS and used at fewer than 16 passages. To establish subcutaneous tumors, 1 × 10^6^ E0771 tumor cells were suspended in PBS and injected into the fourth mammary pad of female B6 mice. Tumor volumes were calculated using the formula V = (width × width × length)/2. For GEM in vivo treatment, 9- to 10-week-old Tg female MMTV-PyMT, E0771 tumor–bearing mice (tumor size between 6–8 mm), or tumor-free mice were treated with GEM (60 mg/kg body weight, Accord Healthcare Inc., diluted in PBS) or PBS control by intraperitoneal (IP) injection 4 times in 2 weeks. For PTX and DOX in vivo treatment, tumor-free mice were treated with PTX (10 mg/kg, IP, MilliporeSigma, catalog T7402, diluted in PBS containing Cremophor EL), DOX (2 mg/kg, intravenous, TOCRIS, catalog 2252, diluted in PBS), or PBS containing Cremophor EL control 3 times in 2 weeks. The mice were sacrificed 2 days after the last treatment to analyze cellularity within BM and lungs. For the experimental metastasis model, E0771-GFP cells (4 × 10^5^) were intravenously injected into B6 WT (anti-Ly6G or anti-CD4/CD8 depletion antibody treated) or CCR2-KO mice pretreated with chemotherapy. The mice were euthanized 2 weeks later after tumor cell injection. To quantify metastatic tumor burden, GFP^+^ tumor cells were quantified by flow cytometry. Lung tissue sections were stained with H&E and scanned with 3DHISTECH’s CaseViewer. Metastasis area was measured using QuPath, and metastasis index was determined by calculation of the percentages of metastasis tumor areas versus total lung areas (76).

### Preparation of lung single-cell suspensions, flow cytometry, and cell sorting.

Lungs were washed extensively in PBS, then minced before digestion with collagenase IV (0.2 mg/mL), DNase I (0.002 mg/mL), and hyaluronidase (10 ng/mL) in complete RPMI 1640 medium (all MilliporeSigma) for 30 minutes at 37°C. After incubation, digestion was immediately stopped by addition of 5 mL cold medium. The suspension was then filtered through a 40 μm cell strainer (Corning) into Petri dishes, and extra tissue chunks were further mashed with syringe columns. The red blood cells were lysed by adding ACK lysis buffer (made in-house) for about 1 minute and washed twice with complete medium. For flow cytometry analysis, the cells were blocked in the presence of anti-CD16/CD32 at 4°C for 10 minutes and stained on ice with the appropriate antibodies and isotype controls in PBS containing 1% FBS. The fluorochrome-labeled antibodies against mouse CD45, CD11b, Ly6G, Ly6C, F4/80, CD4, CD8, IFN-γ, granzyme B, and Foxp3 and their corresponding isotype controls were purchased from BioLegend. APC-conjugated anti-mouse CCR2 antibody was from R&D Systems. Fixable Viability Dye eFluor 780 was from Thermo Fisher Scientific. For intracellular staining, the cells were fixed and permeabilized following surface staining. The samples were acquired using FACSCanto cytometer (BD Biosciences) or Cytek Aurora cytometer and analyzed using FlowJo software. The lung macrophage population (CD45^+^CD11b^hi^F4/80^+^CD11c^−^) was sorted by using BD FACSAria III. Fixable Viability Dye was used to exclude dead cells. An analysis after sorting determined the purity of the macrophages with approximately 90% purity. Antibodies used in flow cytometry are listed in Supplemental Table 1.

### Reactive myelopoiesis analysis.

BM suspensions were prepared by harvesting mouse femur and tibia bones and flushing them with complete medium. The cells were harvested, and red blood cells were lysed by using ACK lysis buffer. The cells were washed twice and suspended in complete medium. For BM stem and progenitor analysis, BM cells were stained with antibodies for lineage markers (CD19, Ter119, CD11b, Ly6G/C, CD3, NK1.1), along with anti-Ly6A/E (Sca1), anti-CD117 (c-Kit), anti-CD48, and anti-CD150 (BioLegend) for the LSK cell population and MPPs. For BM cell in vitro differentiation assay, BM cells were cultured in the presence of recombinant mouse GM-CSF (20 ng/mL) (36) or 20% E0771 CM for 2 days. The culture medium including nonadherent cells was entirely discarded at day 3 and replaced by medium containing GM-CSF or E0771 CM for an additional 4 days. The phenotype of in vitro–cultured cells was analyzed using flow cytometry. Immunosuppression assay of BM Ly6C^+^ cells was performed by coculturing with CFSE-labeled splenocytes from OVA TCR-Tg OT-I mice at a 1:1 ratio in the presence of 20 μg/mL OVA for 3 days (18). For assessment of CFU-GM colony numbers, BM cells (5 × 10^3^) were seeded in MethoCult GF M3534 (STEMCELL Technologies, catalog 03534) methylcellulose-based medium containing recombinant cytokines for mouse myeloid progenitor cells. The colony numbers of CFU-M and CFU-GM with more than 50 cells were determined after 7 days of culture.

### CyTOF mass cytometry data acquisition and data analysis.

Lung cell staining was performed according to the protocol of Maxpar Cell Surface Staining with Fresh Fix (Fluidigm). Prior to acquisition, cells were suspended in a 1:9 solution of cell acquisition solution:EQ 4 element calibration beads (Fluidigm) at an appropriate concentration at no more than 600 events per second. Data acquisition was performed on the CyTOF Helios system (Fluidigm). FCS files were normalized with Helios instrument work platform (FCS Processing) based on the calibration bead signal used to correct any variation in detector sensitivity. CyTOF data analysis was performed with FlowJo software. Total events were gated after removing beads, doublets, and dead cells. tSNE and FlowSOM clustering analysis for CyTOF data were performed using FlowJo Plugins platform. tSNE analysis was performed on all samples combined. Different immune populations were defined by the expression of specific surface markers. Antibodies used in CyTOF are listed in Supplemental Table 2.

### Cytokine array and ELISA.

The cytokine/chemokine profile of mouse plasma or culture supernatants was determined by using Proteome Profiler Mouse Cytokine Array kit (R&D Systems, catalog ARY006). Mouse factor X total antigen was measured using a kit from Molecular Innovations (catalog MFXKT-TOT).

### qRT-PCR.

Small pieces of lung tissue or sorted lung macrophages were frozen in TRIzol (Invitrogen) at −80°C. RNA was extracted and transcribed to cDNA with a reverse transcription kit (Bio-Rad). qRT-PCRs were performed using SYBR Green Supermix (Bio-Rad). We normalized gene expression level to ribosomal protein L13a (*RPL13A*) housekeeping gene and represented data as fold differences by the 2^−ΔΔCt^ method. The primer sequences of real-time PCR were as follows: *F10*: F: 5′-GACAATGAAGGGTTCTGTGG-3′ and R: 5′-CTGTGTTCCGATCACCTACC-3′; *CCL2*: F: 5′-GGTCCCTGTCATGCTTCTGG-3′ and R: 5′-GCTGCTGGTGATCCTCTTGT-3′; *S100A8*: F: 5′- GGAGTTCCTTGCGATGGTGAT-3′ and R: 5′- GTAGACATATCCAGGGACCCAGC-3′; *S100A9*: F: 5′-AGCATAACCACCATCATCGACAC-3′ and R: 5′-TGTGCTTCCACCATTTGTCTGA-3′; *TGFB1*: F: 5′-TGCTAATGGTGGACCGCAA-3′ and R: 5′-CACTGCTTCCCGAATGTCTGA-3′. Each sample was run in duplicate.

### mtROS, mitochondrial membrane potential, and dysfunctional mitochondria measurement.

For quantification of mtROS levels, BM cells were incubated with MitoSOX Red (5 μM, Thermo Fisher Scientific, catalog M36008) at 37°C in PBS and then with appropriate BM progenitor or monocyte markers plus viability dye for 20 minutes at 4°C. The levels of mtROS were determined by flow cytometry. The mitochondrial membrane potential and dysfunctional mitochondria were measured by staining BM cells with TMRM (500 nM, Thermo Fisher Scientific, catalog T668), MitoTracker Green (100 nM, Thermo Fisher Scientific, catalog M7514), and MitoTracker Red (50 nM, Thermo Fisher Scientific, catalog M7512) for 30 minutes at 37°C in PBS and then with appropriate BM progenitor makers plus viability dye for 20 minutes at 4°C.

### RNA-Seq and analysis.

Lung macrophages from PBS- and GEM-treated tumor-free mice were sorted, and RNA was extracted using a QIAGEN RNeasy Kit. The quantity of the purified RNA samples was measured by the RNA High Sensitivity Kit in the Qubit fluorometric quantification system (Thermo Fisher Scientific). Libraries were prepared using the Universal Plus mRNA-Seq with NuQuant (NuGEN). The pooled library was run on MiSeq to test quantity and quality using the MiSeq Reagent Nano Kit V2 300 cycles (Illumina). Sequencing was performed on the Illumina NextSeq 500 using the NextSeq 500/550 75 cycle High Output Kit v2.5. Differential expression was performed using 2 tools, DESeq2 and Cuffdiff2. DEGs at *P* value cutoff 0.01, or *q* value cutoff of 0.01 with log_2_ fold-change of 0 for the pairwise comparisons, were used for further analysis of enriched GO biological processes. A volcano plot was created to examine the distribution of log_2_ fold-change at different significance levels. RNA-Seq data were deposited with National Center for Biotechnology Information Gene Expression Omnibus accession GSE217105.

### Rivaroxaban in vivo treatment.

Rivaroxaban (MilliporeSigma, catalog SML2844) is an orally active, direct inhibitor of FXa. A stock solution was made by dissolving the rivaroxaban in DMSO. After mixing with 0.5% methylcellulose + 0.2% Tween 80, rivaroxaban (20 mg/kg) or solvent control was orally administered to mice daily via oral gavage for 2 weeks.

### Neutrophil, T cell, and macrophage in vivo depletion.

Depletion of neutrophils and CD4^+^ and CD8^+^ T cells was performed by IP injection of anti-Ly6G mAb (300 μg, twice a week, Bio X Cell, catalog BE0075-1), along with anti-CD4 (clone GK1.5, Yan Lab) and anti-CD8 mAbs (clone 2.43, Yan Lab, weekly, 250 μg). Macrophage depletion was performed by IV injection of clodrosome (200 μL/mouse, 3 times per week, Encapsula NanoSciences, catalog CLD-8909). The depletion efficiency was examined by flow cytometry.

### Adoptive transfer of BM monocytes and BM-derived macrophages.

BM cells were harvested from PBS- and GEM-treated tumor-free mice. BM monocytes were purified using mouse BM monocyte isolation kit (Miltenyi Biotec, catalog 130-100-629) and autoMACS Pro Separator (Miltenyi Biotec). The purified monocytes (1.5 × 10^6^) were adoptively transferred into CCR2-KO mice twice, 48 hours apart. For macrophage *F10* gene knockdown, BM-derived macrophages were transfected with *F10* siRNA (Thermo Fisher Scientific, catalog AM16706) and control siRNA (Thermo Fisher Scientific, catalog AM4635) and Lipofectamine RNAiMAX transfection reagent (Thermo Fisher Scientific, catalog 13778030) followed by stimulation with 20% E0771 CM for 24 hours. The control and *F10* gene–knockdown macrophages (2 × 10^6^) were adoptively transferred into CCR2-KO mice twice, 48 hours apart. E0771-GFP cells were IV injected into CCR2-KO recipient mice 48 hours later, and lung metastasis was determined 14–17 days after tumor cell injection.

### Statistics.

Data were analyzed using GraphPad Prism software. An unpaired 2-sided *t* test and 1-way or 2-way ANOVA were used to calculate significance. All graph data are expressed as mean ± SEM. Significance was assumed to be reached at *P* < 0.05.

### Study approval.

All animal experiments were approved by the Institutional Animal Care and Use Committee of the University of Louisville (IACUC 21873).

## Author contributions

CW, QZ, JY, and CD conceptualized and designed the study. CW, QZ, RS, JW, XH, and HL developed the methodology and acquired data. ECR analyzed RNA-Seq data. CW, QZ, JY, and CD analyzed and interpreted data. JY and CD wrote the manuscript and supervised the study. CW initiated this study and so is listed before QZ.

## Supplementary Material

Supplemental data

## Version 1 03/28/2023

In-Press Preview

## Version 2 05/08/2023

Electronic publication
