# Supplementary material for: Reactive myelopoiesis and FX-expressing macrophages triggered by chemotherapy promote cancer lung metastasis
Source: JCI Insight. 2023 May 8;8(9):e167499. doi: 10.1172/jci.insight.167499 (PMC10243818; doi:10.1172/jci.insight.167499)
Supplement: Supplemental data [file jciinsight-8-167499-s070.pdf]

Supplemental Data

Reactive myelopoiesis and FX-expressing macrophages triggered by chemotherapy promote cancer lung metastasis

Caijun Wu, Qian Zhong, Rejeena Shrestha, Jingzhi Wang, Xiaoling Hu, Hong Li, Eric C. Rouchka, Jun Yan, Chuanlin Ding

Supplemental Table 1. Antibodies used in Flow cytometry and Western blotting

Antibodies	Company and catalog number
PerCP/Cyanine5.5 anti-mouse CD45	BioLegend No. 157208
PE/Cyanine7 anti-mouse CD45	BioLegend No. 103114
APC anti-mouse/human CD11b	BioLegend No. 101212
PE anti-mouse Ly6G	BioLegend No. 127608
PerCP anti-mouse Ly6C	BioLegend No. 128028
PE anti-mouse F4/80	BioLegend No. 123110
FITC anti-mouse CD11c	BioLegend No. 117306
APC anti-mouse CD3	BioLegend No. 100236
APC anti-mouse CD4	BioLegend No. 100412
FITC anti-mouse CD8a	BioLegend No. 100706
APC anti-mouse NK-1.1	BioLegend No. 108710
APC anti-mouse CD19	BioLegend No. 115512
APC anti-mouse Ly-6G/Ly-6C	BioLegend No. 108412
APC anti-mouse TER-119	BioLegend No. 116212
Anti-mouse Ly6A/E (Sca-1)	BD Bioscience No. 553108
PE/Cyanine7 anti-mouse CD117 (c-kit)	BioLegend No. 135112
FITC anti-mouse CD48	BioLegend No. 103404
PE/Cyanine5 anti-mouse CD150 (SLAM)	BioLegend No. 115912
PE anti-mouse IFN-γ	BioLegend No. 505808
PE anti-human/mouse Granzyme B	BioLegend No. 372208
PE anti-mouse FoxP3	BioLegend No. 126404
APC-conjugated anti-mouse CCR2	R&D Systems No. FAB5538A-100
Fixable Viability Dye eFluor™ 780	Thermo Fisher Scientific No. 65-0865-14
Polyclonal Rabbit anti-Human F10 / Factor X Antibody	LifeSpan BioSciences No. LS-C331476

Supplemental Table 2. Antibodies used in CyTOF

	**Antibodies**	**Company and catalog number**
1	Anti-Mouse CD45 (30-F11)-89Y	Fluidigm, No. 3089005B
2	Anti-Mouse Ly-6G (1A8)-141Pr	Fluidigm, No. 3141008B
3	Anti-Mouse CD11c (N418)-142Nd	Fluidigm, No. 3142003B
4	Anti-Mouse CD69 (H1.2F3)-143Nd	Fluidigm, No. 3143004B
5	Anti-Mouse CD4 (RM4-5)-145Nd	Fluidigm, No. 3145002B
6	Anti-Mouse F4/80 (BM8)-146Nd	Fluidigm, No. 3146008B
7	Anti-Mouse CD103 (2E7)/148Nd	Biolegend, No. 121402
8	Anti-Mouse CD19 (6D5)-149Sm	Fluidigm, No. 3149002B
9	Anti-Mouse Ly-6C (HK1.4)-150Nd	Fluidigm, No. 3150010B
10	Anti-Mouse CD25 (3C7)-151Eu	Fluidigm, No. 3151007B
11	Anti-Mouse CD3e (145-2C11)-152Sm	Fluidigm, No. 3152004B
12	Anti-Mouse CD274/PD-L1-153Eu (10F.9G2)	Fluidigm, No. 3153016B
13	Anti-Mouse PD-1 (29F.1A12)-159Tb	Fluidigm, No. 3159024B
14	Anti-Mouse CD62L (MEL-14)-160Gd	Fluidigm, No. 3160008B
15	Anti-Human/Mouse CD44 (IM7)-162Dy	Fluidigm, No. 3162030B
16	Anti-Mouse CX3CR1 (SA011F11)-164Dy	Fluidigm, No. 3164023B
17	Anti-Mouse CD8a (53-6.7)-168Er	Fluidigm, No. 3168003B
18	Anti-Mouse CD206/MMR (C068C2)-169Tm	Fluidigm, No. 3169021B
19	Anti-Mouse NK1.1 (PK136)-170Er	Fluidigm, No. 3170002B
20	Anti-Mouse CD11b (M1/70)-172Yb	Fluidigm, No. 3172012B
21	Anti-Mouse CD223/LAG3 (C9B7W)-174Yb	Fluidigm, No. 3174019B
22	Anti-Human/Mouse CD45R/B220 (RA3-6B2)-176Yb	Fluidigm, No. 3176002B
23	Anti-Mouse I-A/I-E (M5/114.15.2)-209Bi	Fluidigm, No. 3209006B
24	Anti-Mouse CD127/IL7Ra (A7R34)-175Lu	Fluidigm, No. 3175006B

Supplemental Figure 1

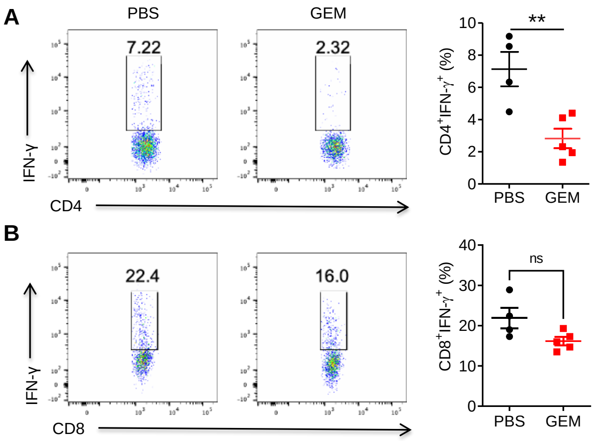

Figure S1. Mice bearing subcutaneous E0771 tumor cells were treated with 4 times of GEM in two weeks. Lung tissues were collected 2 days later after last GEM treatment. Cells were stimulated with PMA/Ionomycin in the presence of protein transport inhibitor brefeldin A for 4 hours. IFN-γ producing CD4 T^+ cells (**A**) and $CD8^+$ T cells (**B**) were determined by intracellular cytokine staining and Flow cytometry. Each dot represents one mouse (n=4-5). ***p <0.01* by unpaired 2-sided *t* test.

Supplemental Figure 2

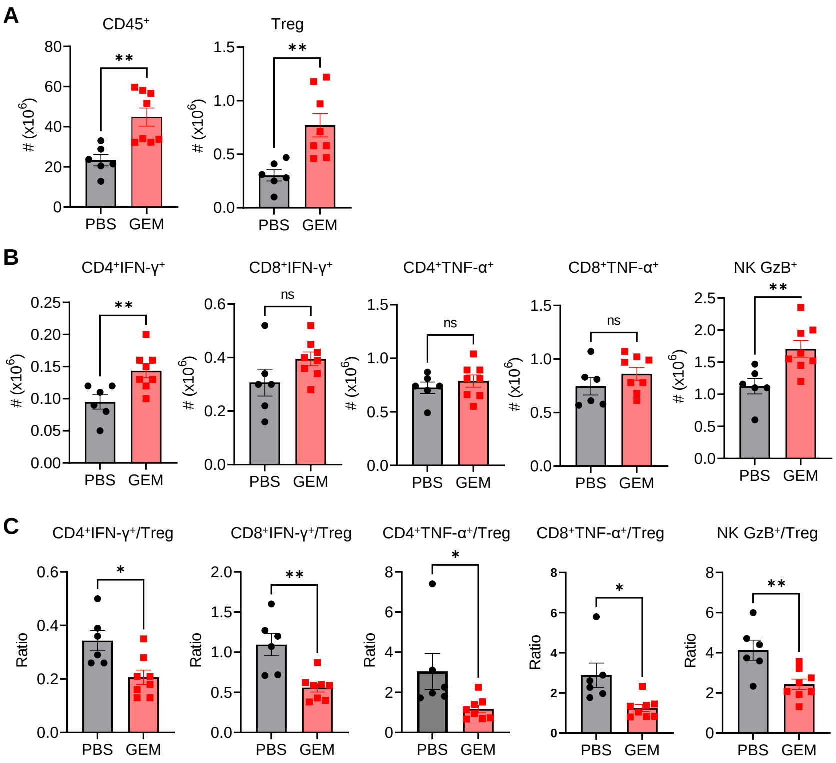

Figure S2. Tumor free mice were treated with 4 times of GEM or PBS followed by intravenous injection of $4x10^5$ E0771-GFP cells. Lung tissues were collected at day 14 after tumor cell injection. (**A**) Total numbers of $CD45^+$ and Treg cells in each lung. (**B**) Total numbers of effective T cells and NK cells. (**C**) Ratios of effective T cells and NK cells to Treg cells. Each dot represents one mouse (n=6-8). *p <0.05, **p <0.01 by unpaired 2-sided t test.

Supplemental Figure 3

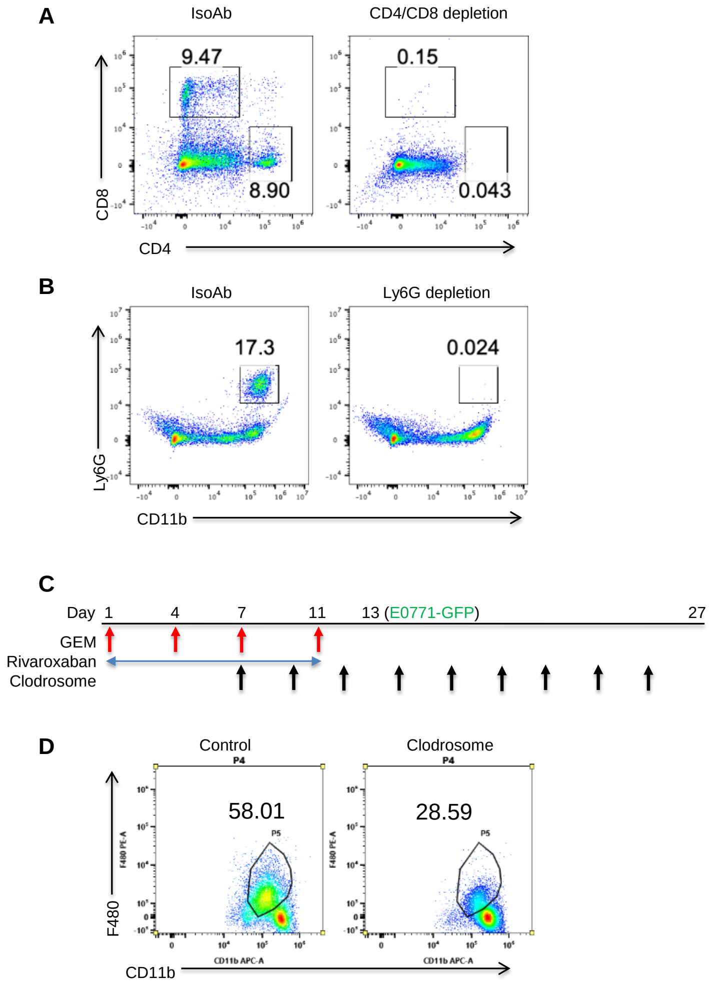

Figure S3. (**A**) Depletion efficiency of CD4$^+$ and CD8$^+$ T cells in the lungs. (**B**) Depletion efficiency of Ly6G$^+$ cells in the lungs. (**C**) Schema for in vivo GEM and Rivaroxaban treatment, macrophage depletion, and E0771-GFP tumor cell intravenous injection. (**D**) Lung macrophage depletion efficacy after intravenous injection of 3 times of Clodrosome was determined by staining cells with CD45, CD11b, F4/80, and viability dye. Cells were gated on CD45$^+$CD11b$^+$ population.

Supplemental Figure 4

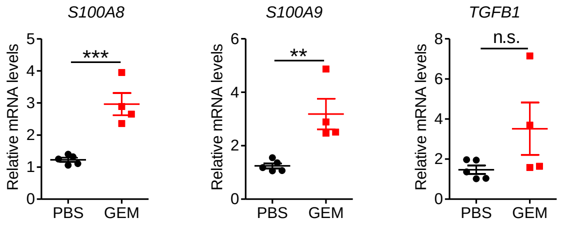

Figure S4. Naïve mice were treated with 4 times of GEM in two weeks. Lung tissues were collected 48 hours later after last treatment. Gene expression of *S100A8*, *S100A9*, and *TGFB1* in lung tissues from GEM and PBS treated mice was determined with qRT-PCR. Each dot represents one mouse (n=4-5). **p <0.01, ***p <0.001 by unpaired 2-sided t test.

Supplemental Figure 5

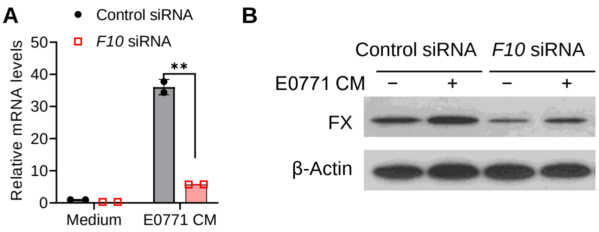

Figure S5. BM-derived macrophages were transfected with control siRNA and *F10* siRNA. 24 hours later cells were treated with 20% E0771 conditioned medium (CM) for 24 hours. *F10* gene knock down efficiency was determined by using qRT-PCR (**A**) and Western blotting (**B**). **p <0.01 by unpaired 2-sided t test.

Supplemental Figure 6

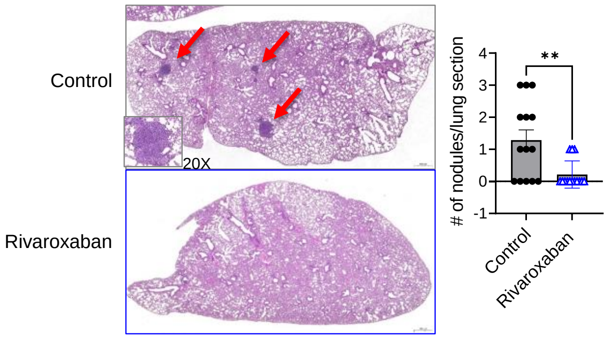

Figure S6. E0771-GFP subcutaneous tumor-bearing mice (primary tumor size between 6-8 mm in diameter) were treated with Rivaroxaban (oral, 20 mg/kg, daily) or solvent control for two weeks. Lung tissues were collected at day 31 after tumor cell injection. The metastasis in lungs was determined by evaluation of tumor nodules using H&E staining. One section represents one mouse (n=14). Numbers of nodule per lung section were counted and summarized. $^{**}p < 0.01$ by unpaired 2-sided t test.

Supplemental Figure 7

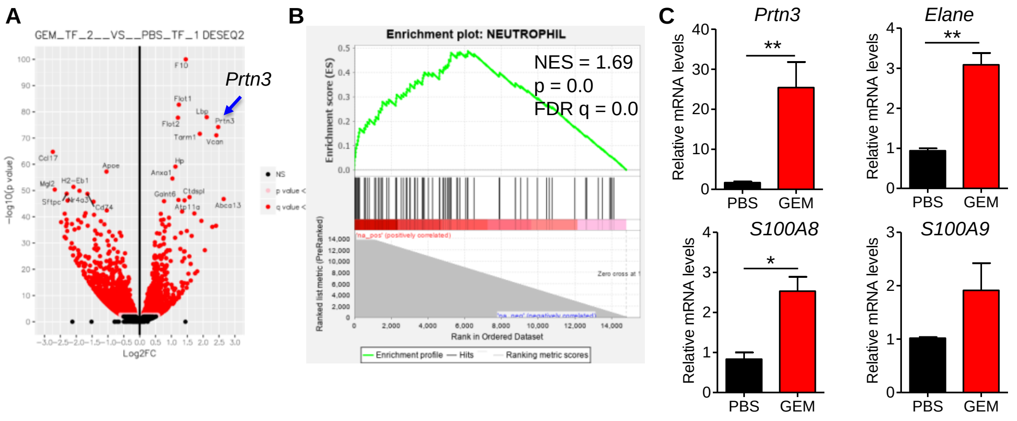

Figure S7. (**A**) Differential gene expression in sorted macrophages from GEM-treated tumor-free mice as compared to macrophages from PBS-treated mice. Gene *Prtn3* is one of DEGs in lung macrophages. (**B**) Analysis of lung macrophage RNA-seq data revealed the enrichment plot with upregulation of neutrophil signature genes in lung macrophages from GEM-treated versus PBS-treated tumor-free mice. (**C**) Lung monocytes ($CD45^+CD11b^+Ly6C^{high}$) were sorted for gene expression analysis using qRT-PCR. **p* <0.05, ***p* <0.01 by unpaired 2-sided *t* test.

Supplemental Figure 8

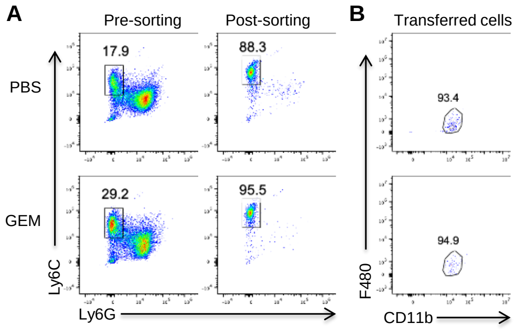

Figure S8. (**A**) Percentages of BM monocytes from PBS and GEM treated mice before and after purification. (**B**) CFSE labeled purified monocytes ($1.5x10^6$) were intravenously injected into CCR2 KO mice. Expression of CD11b and F480 in transferred cells ($CFSE^+$) in the lungs analyzed 48 hours later.

Supplemental Figure 9

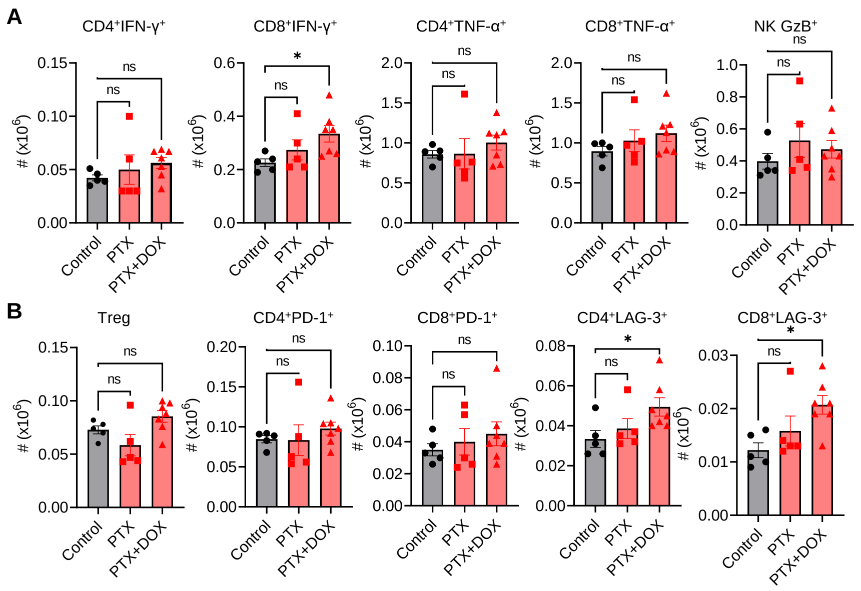

Figure S9. Tumor free mice were treated with PTX or PTX plus DOX. Lung tissues were collected 48 hours later. (**A**) Total numbers of effective $CD4^+$, $CD8^+$ T cells and granzyme B $(GzB)^+$ NK cells. (**B**) Total cell number of immunosuppressive Treg and PD-1, LAG-3 expressing $CD4^+$, $CD8^+$ T cells. $*p < 0.05$ by ordinary one-way ANOVA.
